# Molecular Landscape of Small Bowel Adenocarcinoma

**DOI:** 10.3390/cancers14051287

**Published:** 2022-03-02

**Authors:** Karan Pandya, Michael J. Overman, Pat Gulhati

**Affiliations:** 1 Division of Medical Oncology, Department of Medicine, Robert Wood Johnson Medical School, Rutgers University, New Brunswick, NJ 08901, USA; karan.pandya@rutgers.edu; 2 Rutgers Cancer Institute of New Jersey, Rutgers University, New Brunswick, NJ 08901, USA; 3 Department of Gastrointestinal Medical Oncology, Division of Cancer Medicine, MD Anderson Cancer Center, Houston, TX 77030, USA; moverman@mdanderson.org

**Keywords:** small bowel adenocarcinoma, BRAF alteration, ERBB2/HER2 alteration, microsatellite instability (MSI), tumor mutational burden (TMB), colorectal cancer (CRC), gastric cancer (GC)

## Abstract

**Simple Summary:**

Small bowel adenocarcinoma (SBA) is a rare malignancy with worse prognosis compared to other cancers of the gastrointestinal tract. Over 90% of SBA tumors harbor targetable genetic alterations. Molecular analysis to identify these alterations, using tissue- or blood-based next generation sequencing, is critical and may impact treatment decisions. The aim of our review is to highlight molecular drivers of SBA tumorigenesis. We highlight key mutational and transcriptomic differences between SBA and colorectal cancer, from which much of the clinical management of SBA is currently extrapolated. We provide evidence that SBA is a molecularly unique intestinal malignancy, with distinct genomic alterations predictive of response to targeted therapy and immunotherapy.

**Abstract:**

Small bowel adenocarcinoma (SBA) is a rare malignancy, with lower incidence, later stage at diagnosis, and poor overall prognosis compared to other cancers of the gastrointestinal tract. Owing to the rarity of the disease along with the paucity of high-quality tissue samples and preclinical models, little is known about the molecular alterations characteristic of SBA. This is reflected by the fact that the clinical management of SBA is primarily extrapolated from colorectal cancer (CRC). Recent advances in genomic profiling have highlighted key differences between these tumors, establishing SBA as a molecularly unique intestinal cancer. Moreover, comprehensive molecular analysis has identified a relatively high incidence of potentially targetable genomic alterations in SBA, predictive of response to targeted and immunotherapies. Further advances in our knowledge of the mutational and transcriptomic landscape of SBA, guided by an increased understanding of the molecular drivers of SBA, will provide opportunities to develop novel diagnostic tools and personalized therapeutic strategies.

## 1. Introduction

Small intestinal cancer is a rare cancer [1,2], with few prospective studies published to date to guide clinical management. Moreover, owing to its rarity, clinical trial options for those newly diagnosed with small bowel cancer are limited, consequently leading to a relative dearth of diagnostic, predictive, and prognostic biomarkers [3]. Although the small intestine makes up 75% of the digestive tract, only 3% of total digestive cancers arise from the small bowel [4,5]. In 2021, 11,390 new cases of small bowel cancer were estimated, with 2100 cancer-related deaths [5]. Amongst the primary tumors, approximately 60% arise in the duodenum, 25–29% arise in the jejunum, and 10–13% arise in the ileum [2]. When subdivided by histology, carcinoid tumors account for 39–45% of all small intestinal cancers, whereas adenocarcinoma constitutes 31–40% of cases [2].

Interestingly, despite constituting over 90% of the surface area of the gastrointestinal tract, the incidence of SBA is about 50 to 100-fold less than CRC [5,6,7]. Rapid turnover time of small intestinal cells not allowing for the accumulation of genetic defects and rapid transit time through a dilute and alkaline environment with greater lymphoid infiltrate have both been proposed as potential explanations for the relative rarity of SBA [1,8,9,10,11,12]. The etiology of SBA remains largely unknown. Small retrospective analyses have identified cigarette smoking and alcohol as potential environmental risk factors [13,14]; other studies have suggested an association between high sugar or high red meat intake and the development of SBA [4,15,16,17,18]. Several genetic cancer syndromes, namely hereditary nonpolyposis colorectal carcinoma (HNPCC), familial adenomatous polyposis (FAP), and Peutz-Jeghers syndrome, can predispose individuals to SBA development. A number of registry studies and case series have additionally implicated proinflammatory conditions, such as inflammatory bowel disease (IBD) and celiac disease, in SBA carcinogenesis [19,20,21,22].

Amongst patients diagnosed with SBA, intermittent abdominal pain remains the most common presenting symptom [23,24]. Whether due to such a non-specific clinical presentation, or due to delay or difficulty with imaging the small bowel, SBA tends to be diagnosed at a later stage compared to CRC, with most patients presenting with lymph node involvement or distant metastasis [8,25,26]. Despite these differences and being associated with inferior overall outcomes, SBA is currently treated in a similar manner to CRC [1,2]. The National Comprehensive Cancer Network (NCCN) recently published the first guidelines specific for SBA [27]. While the rarity of this disease and paucity of prospective studies published to date are partially to blame, the lack of molecular knowledge to help guide clinical management has represented a major roadblock. Recent advances in genomic profiling have highlighted differences between SBA and other gastrointestinal neoplasms, establishing SBA as a molecularly unique intestinal cancer. Moreover, comprehensive genomic analysis has identified genetic alterations, predictive of response to targeted and immunotherapies and portentous for future novel pathways for treatment.

## 2. Molecular Characteristics

**Figure 1:**
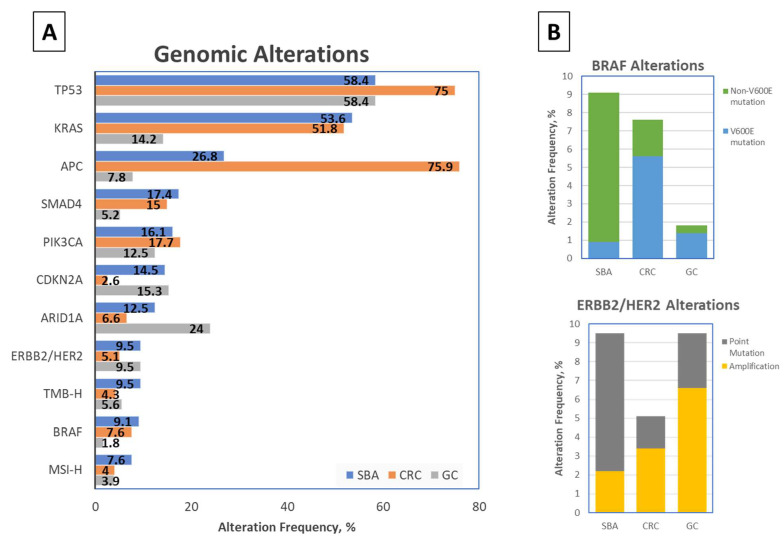
Frequency of genomic alterations in Small Bowel Adenocarcinoma (SBA), Colorectal Cancer (CRC), and Gastric Carcinoma (GC) [1]. (**A**) Genomic alterations noted in >7% of SBA patients, with corresponding genomic alteration frequencies in CRC and GC patients [1]. (**B**) BRAF (above) and ERBB2/HER2 (below) alteration frequencies in SBA, CRC, and GC cohorts, by type of alteration [1].

Genomic profiling studies have identified a number of critical molecular drivers in the pathogenesis of SBA (Figure 1A), including E-cadherin, KRAS, TP53, and SMAD4, among others [3,8,28]. An 18 cancer-related gene panel in 24 SBA cases identified TP53 (54%), KRAS (42%), and APC (11%) genomic alterations as most common [29]. In another 83 patients, a 46-gene panel study reported KRAS (43%), TP53 (41%), APC (13%), SMAD4 (10%), PIK3CA (8%), and ERBB2/HER2 (6%) as the most common genomic alterations [30]. A pivotal study of 7559 patients undergoing genomic sequencing on a 236 or 315 cancer-related gene panel demonstrated that the most common genomic alterations in SBA were TP53 (58.4%), KRAS (53.6%), APC (26.8%), SMAD4 (17.4%), PIK3CA (16.1%), CDKN2A (14.5%), and ARIDIA (12.3%) [1]. Of the 317 SBA tumors profiled in this study, 191 samples were from the primary small bowel, whereas 126 were from metastatic sites—similar rates of genomic alterations per gene were observed in primary vs. metastatic biopsies tested, with a median of 5 genetic alterations per SBA tumor tested [1].

SBA has also been associated with a higher likelihood of microsatellite instability (MSI) and high tumor mutational burden (TMB) [30,31]. In the aforementioned largest SBA genomic profiling study to date, 7.6% of SBA tumors were MSI-high, and 9.5% had high TMB [1]. Moreover, there appears to be a higher rate of mismatch repair (MMR) deficiency in early-stage SBA tumors, up to 23% as shown in a recent study of 63 SBA patients, which included a large cohort of patients with early-stage disease [32]. Over half of the patients with MMR deficient tumors in this study were ultimately found to have underlying Lynch syndrome, an autosomal dominant disorder with germline mutations in DNA MMR genes. Similarly, those with germline inactivation of the APC gene, associated with familial adenomatous polyposis (FAP), have a 4.5% lifetime risk of developing SBA [33,34,35], while those with an inherited STK11 mutation, resulting in Peutz-Jeghers syndrome (PJS), have a relative risk of 520 for developing SBA [36].

Perhaps the finding of greatest clinical relevance through comprehensive genomic analyses is that over 90% of SBAs harbor targetable genetic alterations [1]. Of these potentially actionable alterations, PIK3CA (16.1%), ERBB2/HER2 (9.5%), BRAF (9.1%), ATM (7.6%), FBXW7 (6.9%), and ERBB3 (6.3%) have been most commonly detected, while MEK1 mutations (2.8%), EGFR alterations (2.5%), and activating tyrosine kinase rearrangements [ALK, ROS1, and FGFR2] (0.9%) were less frequently seen in SBA samples [1]. For such a rare malignancy without current FDA-approved treatments and limited clinical management guidelines, gaining insight into these unique molecular characteristics of SBA may provide clues about identifying matched targeted therapies for each genomic alteration.

### 2.1. Comparison with Neighboring Intestinal Cancers

Due to the anatomic proximity, the majority of SBA treatment paradigms have been extrapolated from CRC [1,2]. A study of 85 patients with adenocarcinomas originating from the stomach, small bowel, and colorectum compared chromosomal copy number alterations by primary tumor site, ultimately demonstrating greater overlap between SBA and CRC copy number profiles than between SBA and gastric adenocarcinoma copy number profiles [37].

**Figure 2:**
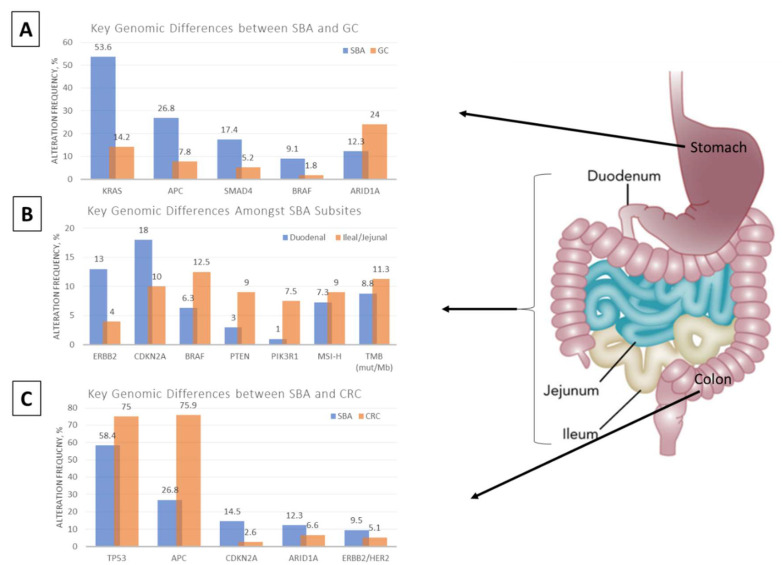
Major differences in genomic alteration frequencies between SBA and GC (**A**), SBA subsites (**B**), and SBA and CRC (**C**) [1]. Adapted with permission form [2].

Despite clustering with CRC and, to a less extent, gastric cancer, SBA remains a unique molecular entity with distinct differences (Figure 2A,C). A recent comparative analysis highlighted genomic alteration differences between SBA and its neighboring cancers as detected from a 236 or 315 cancer-related gene panel [1]. Of the top 20 most commonly altered genes in this study, 12 genes were statistically different between SBA and CRC, and 12 genes were statistically different between SBA and gastric adenocarcinoma [1].

When comparing between SBA and CRC, the most notable differences were seen in APC, TP53, and CDKN2A mutations [1]. APC alteration rates represent one of the most fundamental genomic differences between SBA and CRC. APC gene mutations were far less prominent in SBA (~30% incidence) compared to CRC (~70% incidence). Multiple smaller studies show an even greater variation in frequency of APC gene mutations between SBA and CRC, demonstrating 7–13% reported rate in sporadic SBA as compared to >80% reported rate in CRC [29,30,38,39,40,41]. However, despite having a lower APC mutation rate as compared to CRC, SBA tumors have higher overall total mutation rates, with a few case series’ reporting a greater number of atypical BRAF mutations and ERBB2 point mutations in SBA [1,30,31,42]. Albeit rare in gastric cancers, BRAF mutations were seen at similar rates between SBA and CRC; however, unlike CRC where the majority of BRAF alterations were V600E mutations, most BRAF-altered SBAs harbored inactivating non-V600E mutations [1,2,31]. Moreover, while ERBB2 alterations were largely amplifications in both gastric and colorectal cancers, ERBB2 point mutations were the most frequent type of alterations in SBA [1,2,30,42].

When comparing molecular alteration differences between SBA and gastric carcinoma, variation in the APC mutation rate was also striking, with one study demonstrating 27% incidence of APC mutations in SBA as compared to just 8% in gastric adenocarcinoma [1]. The incidence of KRAS and SMAD4 genetic alterations, which occurred at similar rates with SBA and CRC, was also much higher in SBA with rates of 54% and 17% respectively in one study as compared to 14% and 5% incidence in gastric adenocarcinoma [1]. Meanwhile, TP53 and CDKN2A gene mutations, which occurred more frequently in SBA than CRC, were seen at similar rates between SBA and gastric adenocarcinoma [1].

### 2.2. Small Bowel Subsite Comparison

The small intestine is anatomically subdivided into the duodenum, jejunum, and ileum. While primary tumors of the small intestine may originate from any of these subsites, they are often grouped together owing to their relatively lower incidence compared to other gastrointestinal tumors. Despite this grouping, however, key differences exist (Figure 2B). Histologically, adenocarcinomas represent approximately 59% of duodenal cancers, while carcinoid tumors represent 57% of ileal cancers [2,3]. Moreover, patients with duodenal adenocarcinoma have a higher rate of locoregional failure following curative resection compared to patients with jejunal and ileal adenocarcinoma, suggesting local anatomic differences and subsite-specific molecular alterations driving worse patient outcomes [43]. Recent studies have sought to provide an understanding of differences in molecular alterations across various small bowel subsites.

Prospective genomic profiling of 317 small bowel adenocarcinomas demonstrated similar molecular alteration rates across the small bowel, with no statistical differences seen amongst TP53, KRAS, APC, SMAD4, and PIK3CA genes, which were the top five most commonly altered genes [1]. However, when comparing duodenal adenocarcinomas to cancers of other small bowel sites, both CDKN2A (18% vs. 10%) and ERBB2 (13% vs. 4%) alteration rates were higher in duodenal adenocarcinomas [1]. Another study of 83 patients confirmed these findings, reporting increased ERBB2 mutation rate in duodenal adenocarcinomas (15.8%) compared to jejunal and ileal adenocarcinomas (2.2%) [30]. Conversely, duodenal adenocarcinomas had fewer BRAF (6.3% vs. 12.5%), PTEN (3% vs. 9%), and PIK3R1 (1% vs. 7.5%) gene alterations, as well as lower MSI-high rates (7.3% vs. 9%) and lower overall TMB (8.8 mutations/Mb vs. 11.3 mutations/Mb) compared to other small bowel subsites [1]. Altogether, these findings support the notion of molecular heterogeneity not only between SBA and neighboring adenocarcinomas of the stomach and colorectum, but also across different small bowel subsites.

### 2.3. Comparison across SBA Etiologies

Several hereditary cancer syndromes and proinflammatory conditions have been implicated as risk factors for SBA. Hereditary nonpolyposis colorectal cancer (HNPCC), or Lynch syndrome, is an autosomal dominant germline mutation in DNA mismatch repair (MMR) genes, including MLH1, MSH2, MSH6, PMS2, EPCAM, and PMS1, associated with a 1% lifetime risk of developing SBA [44,45,46,47]. A molecular analysis study of 63 SBA tumors showed MMR deficiency (dMMR) in 14 tumors with confirmed Lynch syndrome in 9 of 14 cases, suggesting that the higher frequency of dMMR seen in SBA as compared to CRC may be explained by a higher frequency of Lynch syndrome in SBA patients, and that the etiology for MSI-high appears to be more commonly related to Lynch syndrome in SBA as opposed to MLH-1 promoter hypermethylation in CRCs [32].

Germline inactivation of the APC gene can result in the formation of hundreds of colonic polyps, a condition known as familial adenomatous polyposis (FAP). Although it is more typically associated with early-onset CRC, individuals with FAP have a 4.5% lifetime risk of developing SBA [33,34,35]. Once FAP patients undergo colectomy for risk reduction of CRC, surveillance for SBA becomes increasingly important.

A small subset of patients who develop SBA have underlying inflammatory bowel disease (IBD), particularly Crohn’s disease, or celiac disease. One study showed that patients with Crohn’s disease carried a cumulative 0.2% risk at 10 years and 2.2% at 25 years of developing SBA [48], translating into a 60-fold increased risk [49,50]. Interestingly, APC mutations appear to be exclusively noted in non IBD-associated SBA patients [1]. Instead, IBD-associated SBA has a higher frequency of CDKN2A/B, CASP8, and ATRX mutations [1], as well as an increased predilection for the ileum and significantly shorter overall survival as demonstrated in a 37 IBD-associated SBA patient study [51]. Meanwhile, several registry studies and case series’ have conferred a 34-fold increased risk of developing SBA amongst patients with celiac disease [19,20,21,22]. Studies of celiac disease-related SBA have demonstrated particularly high rates of microsatellite instability, ranging from 50–73% [31,52,53]. In one study of 15 patients with celiac disease-associated SBA, 10 patients had MSI-high tumors. Of note, each of these 10 cases had MLH-1 promoter hypermethylation, implicating a causal role of hypermethylation in celiac disease-associated SBA, and suggesting the potential for incorporating immunotherapy in its management [52].

## 3. Potentially Targetable Genomic Alterations

**Figure 3:**
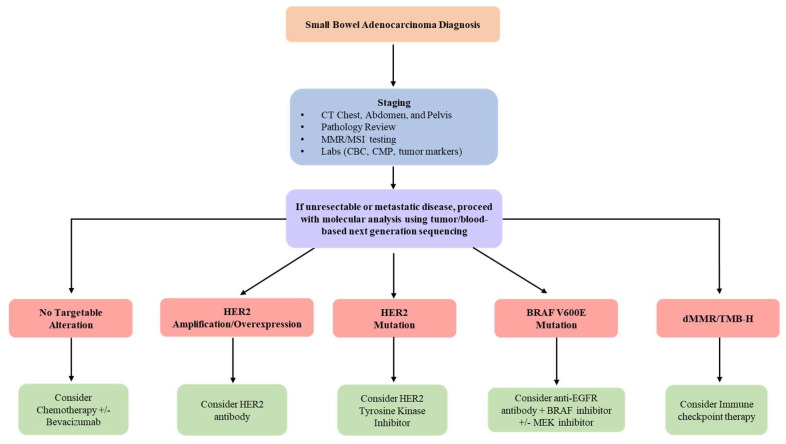
Flow diagram outlining management with incorporation of molecular analysis to determine the treatment plan for unresectable/metastatic SBA patients.

Molecular characterization efforts have uncovered numerous targetable alterations, as well as higher rates of MSI and increased TMB in patients with SBA. In one large study including 317 SBA patients, potentially targetable genomic alterations were seen in greater than 90% of cases [1]. These targetable alterations provide additional therapeutic options and suggest an increased role for the use of targeted therapy and immunotherapy in the future management of SBA (Figure 3).

### 3.1. BRAF

In addition to key differences in the genomic alteration patterns amongst the various small bowel subsites and between neighboring intestinal cancers, the type of molecular alterations seen in SBA add to its uniqueness. In the previously mentioned comparative analysis of 317 SBA tumors and 6353 colorectal tumors, BRAF alterations were identified at similar rates (9.1% of SBA cases vs. 7.6% of CRC cases) [1]. However, there was significant variation in the type of alteration (Figure 1B). While 73% of BRAF mutations in CRC were the canonical BRAF V600E (class I, RAS-independent), this codon was infrequently altered in SBA, representing just 10.3% of BRAF alterations [1]. Another analysis of 106 SBA cases identified 11 BRAF mutated tumors but none of these were BRAF V600E mutated [31]. Instead, most BRAF mutations seen in SBA patients appear to be inactivating mutations (class III, RAS-dependent) [1]. Of the 29 BRAF-mutated SBAs identified in one study, 16 tumors were class III BRAF mutated, associated with impairment of kinase function [1]; meanwhile, just 3 of 29 BRAF-mutated SBA tumors had class I BRAF V600E mutations, while another seven were class II RAS-independent BRAF activating non-V600E mutations [1]. Interestingly, amongst SBA tumors specified as duodenal adenocarcinoma, there appears to be lower frequency of BRAF alterations compared to jejunal or ileal SBAs [1]. Feedback reactivation of EGFR that in turn activates MAPK via CRAF and RAS is a mechanism of resistance to BRAF inhibitors in CRC, and it is through this mechanism that most of these atypical BRAF mutations are still felt to be oncogenic, and as such potentially targetable using pan-RAF or MEK inhibition [54] (Figure 3).

### 3.2. ERBB2/HER2

In addition to atypical BRAF mutations, ERBB2/HER2 genomic alterations are also different when comparing duodenal SBAs to jejunal/ileal SBAs, and when comparing SBAs to gastric and colorectal adenocarcinomas (Figure 1B). Amongst SBA tumors, those identified as duodenal adenocarcinomas harbor significantly increased ERBB2/HER2 alteration frequency (*p* < 0.01) compared to other SBA tumors [1]. In one study, 12% of all SBAs (10 of 83 cases) harbored ERBB2/HER2 alterations [30]. Another study of 317 SBA patients identified a similar ERBB2/HER2 alteration rate of 9.5% [1]. In this study, ERBB2/HER2 alteration rates were similar to the gastric cancer cohort (9.5% genomic alteration rate), however 69% of these alterations were amplifications in gastric cancer, as opposed to just 23% in SBAs [1]. Additionally, colorectal adenocarcinomas had a decreased ERBB2/HER2 alteration rate of 5.1%, but had a similar percentage (66%) of ERBB2 amplifications as gastric adenocarcinomas [1]. Meanwhile, although ERBB2 alterations are present in SBAs, these alterations appear to be primarily point mutations. Of the 317 SBA tumors analyzed in the one study, 26 cases (8.2% of the total) had activating ERBB2 mutations, while 7 cases (2.2% of the total) had ERBB2 amplifications, and three cases had both ERBB2 amplification and point mutation [1]. In three other smaller cohorts, 70% to 76% of the ERBB2/HER2 genomic alterations identified were reported as mutations [30,31,42]. Preclinical in-vitro and in-vivo models of SBA with ERBB2 kinase activating mutations have been developed to test for sensitivity to ERBB2 tyrosine kinase inhibitors, with reduction in tumor growth up to 59% after dacomitinib treatment [42]. These findings have important therapeutic implications for SBA patients harboring ERBB2/HER2 alterations (Figure 3).

### 3.3. Microsatellite Instability and Tumor Mutational Burden

A number of studies have reported higher rates of microsatellite instability-high (MSI-H) or dMMR in SBAs, as compared to neighboring gastrointestinal tract tumors. In an 83-patient case series, 21.6% of patients had MSI-H/dMMR tumors [30], while in another 106-patient case series, 14.2% of patients had MSI-H/dMMR tumors [31]. While higher rates of MSI-H/dMMR SBAs are particularly seen in those with earlier stage disease [32], even studies with primarily advanced-stage SBA patients showed increased rates of microsatellite instability as compared to CRC and gastric cancer. In the largest-scale genomic comparison of SBA to gastric and colorectal cancers to date, of the 170 primarily advanced-stage SBA tumors for which MSI status was assessed, 13 (7.6%) tumors were MSI-H, as opposed to 4% and 3.9% rates respectively in the colorectal cancer and gastric cancer cohorts [1]. All 13 SBA MSI-H cases had at least one mismatch repair gene inactivating alteration, with 10 of 13 cases involving MSH2, MSH6, or MLH1 gene loss of function [1]. Of note, MSH6 genomic alterations were 2-fold greater in SBA as compared to gastric cancer or CRC, with all three cancer cohorts demonstrating very rare POLE alterations [1].

Furthermore, 9.5% of the 317 SBA tumors analyzed had high TMB, defined as greater than 20 mutations per megabase (Mb), as compared to just 4.3% of CRC cases and 5.6% of gastric cancer cases [1]. All MSI-H cases in each of the three cohorts had TMB greater than 10 mutations/Mb [1]. Interestingly however, while microsatellite stable (MSS) SBA tumors generally tended to have a much lower TMB, with two studies reporting median TMB of 4.2 and 4.3 mutations/Mb [1,31], there were a subset of SBA MSS tumors which also had a high TMB [1]. Amongst SBAs, albeit not statistically significant, jejunal/ileal adenocarcinomas tend to have higher MSI-H rates than duodenal SBAs, with a correspondingly higher overall TMB (11.3 mutations/Mb as compared to 8.8 mutations/Mb in duodenal SBAs), a result that was statistically significant [1].

Anti-programmed cell death 1 (PD-1) and anti-cytotoxic T-lymphocyte-associated protein 4 (CTLA4) checkpoint inhibitors have known efficacy in MSI-H cancers, as well as TMB-high cancers [55,56]. Given the greater incidence of both MSI-H SBAs and TMB-high SBAs than their gastric or colorectal cancer counterparts, routine assessment for MSI-H/dMMR should be conducted in all SBAs and may ultimately prove critical to predicting response to immune checkpoint therapies (Figure 3).

### 3.4. EGFR

In one study, SBAs and CRCs had similar 2.5% rates of EGFR alterations, while a 4% EGFR alteration rate was seen in the gastric cancer cohort [1]. Of these EGFR alterations, amplification of EGFR was less frequent in SBA and CRC (1.6% of cases each) as compared to gastric cancer (3.4% of cases) [1]. The role for EGFR therapy in RAS wild-type CRC has been widely investigated, with drugs such as cetuximab and panitumumab gaining FDA approval [57]. The role for anti-EGFR directed therapy in RAS wild-type SBA is less clear. A number of case reports have suggested clinical benefit with EGFR directed therapy, either alone or in combination with chemotherapy [58,59,60], whereas one clinical trial involving 9 SBA patients receiving single agent panitumumab demonstrated no responses [61]. The uncertain benefit of anti-EGFR agents in SBA, as well as the differential benefit seen with this therapy in left-sided versus right-sided CRC, has been hypothesized to be related to the embryological and anatomic development of the gastrointestinal tract—the midgut ultimately develops into much of the small intestine as well as the proximal colon, while the more distal, left-sided colon is derived from the hindgut.

### 3.5. Other Targetable Genomic Alterations

Targeted alterations in several additional genes, including PIK3CA, MEK1, APC and other wingless integration site family member (WNT) pathway genes, and receptor tyrosine kinase fusions have also been described in SBA. PIK3CA represents the most common potentially targetable genomic alteration in SBA, occurring in 51 of 317 (16.1%) patients in one study [1]. Of these 51 patients, 21 had activating mutations in exon 9, while 15 had activating mutations in exon 20 [1], revealing two possible therapeutic targets. Similar rates of PIK3CA genomic alterations have been reported in CRC and gastric cancers (17.7% and 12.5% respectively) [1]. Phosphoinositide 3-kinase (PI3K) inhibition has been clinically validated, resulting in FDA approved therapies for the hematologic malignancies and most recently breast cancer; molecular characterization efforts suggest PI3K inhibition may benefit a subset of SBA patients.

Genomic alterations of the mitogen-activated protein kinase (MAP2K1 or MEK1) gene were much less frequent than PIK3CA alterations, seen in just 2.8% of SBAs [1]. However, there may be a role for MEK inhibitors in this small cohort of SBA patients. Similarly, 3 of 317 SBA cases had activating receptor tyrosine kinase rearrangements identified, involving ALK, ROS1, and FGFR2 genes [1]. Another recent study found a fusion of the intestinal stem-cell marker olfactomedin 4 (OLFM4) and the proto-oncogene RET in an SBA patient [62]. OLFM4 expression was found to be frequently diminished in SBA, with targeted OLFM4-RET expression leading to development of adenocarcinoma in mouse models [62]. Based on this study, OLFM4-RET may serve as an additional oncologic driver of small intestinal carcinogenesis, implicating a future role for treatment with RET kinase inhibitors in SBA patients harboring OLFM4-RET fusions.

Activation of the WNT pathway through accumulation of beta-catenin may have a role in a subset of SBAs, but this accumulation appears much less frequently caused by inactivating APC gene mutations when compared to CRC [40], as evidenced by the discrepancy in APC genomic alteration rates seen. Interestingly, genomic alterations in other WNT pathway genes were largely mutually exclusive from APC, but also seen relatively uncommonly even in the APC wild-type SBA cohort [1], suggesting a potentially lesser role of the WNT pathway in SBA tumorigenesis as compared to CRC. Nonetheless, with over 1 in 4 SBA patients harboring APC gene alterations, there is no doubt that exploring WNT signaling pathway directed therapies could lead to a significant shift in SBA treatment paradigms. RING finger protein 43 (RNF43) is an E3 ubiquitin ligase with demonstrated effect as an inhibitor of WNT signaling [63,64,65,66,67,68,69]; studies have suggested its role in tumor suppression, by blocking the WNT pathway downstream of oncogenic mutations that activate the pathway [65]. Endogenous RNF43 has been detected in human intestinal crypts, with WNT pathway-mediated tumorigenesis observed in gastrointestinal cancer cells expressing RNF43 mutations [65]. As such, small-molecule inhibitors of the membrane-bound O-acyltransferase Porcupine have been developed to block WNT signaling [67]; in RNF43-mutated small intestinal tumors, which lack the downstream WNT pathway regulation generally provided by RNF43, preclinical models have shown an effect in both CRC and SBA tumor growth suppression [68,69].

### 3.6. Epigenetic Alterations and Non-Coding RNA

While detailed analyses of the relationship between epigenetic and genomic alterations contributing to CRC and GC have been described [70], few studies have investigated these interactions in SBA. DNA hypermethylation is an epigenetic phenomenon frequently implicated in the pathogenesis of gastrointestinal cancers, and in particular, the CpG island methylator phenotype (CIMP) has become increasingly recognized [71,72,73,74,75,76]. CIMP is associated with MLH1 methylation, decreased MLH1 expression, and MSI-H in CRC and GC [71,77,78], as well as with BRAF mutations in CRC [79]. A study of 37 primary SBA tumors, stratified by CIMP, RAS/BRAF mutation status, microsatellite status, and chromosomal instability (CIN) sought to characterize this interrelationship between epigenetic and genomic alterations in SBA [75]. Among the 37 SBA tumors analyzed, 11 displayed high-level CIMP (CIMP-H). CIMP-H with MLH1 methylation was especially common in MSI-H SBA and microsatellite and chromosomally stable (MACS) SBA [75], suggesting similarities with CRC [80,81,82,83]. Meanwhile, an inverse correlation was seen between aberrant methylation and SBA with CIN [75], as described in CRC [84,85]. Approximately 50% of the SBA tumors analyzed were CIN, characterized by frequent KRAS mutation and low-level methylation [75], while the remaining SBA tumors were MSI-H and MACS, characterized by the lack of KRAS mutations and greater incidence of high-level methylation and BRAF mutations [75], implicating two molecularly distinct SBA subtypes with unique tumorigenesis pathways.

Histone modification is another mechanism of epigenetic alteration well studied in CRC [86,87,88,89,90], which also appears to play a role in SBA pathogenesis. A study of 17 SBA tumor samples undergoing next generation sequencing identified truncation mutations upstream of the SET domains of two histone-lysine N-methyltransferases, KMT2C and KMT2D, in 70% and 18% of SBA samples [42], substantially greater than the 10% alteration rate seen in CRC [91,92,93]. These genes, in addition to the zinc finger gene, suppressor of zeste 12 homolog (SUZ12), which was mutated in one third of SBA samples analyzed [42], play an integral role in the histone methylation process, and have been implicated in esophageal and prostate tumorigenesis [94,95,96,97]. KMT2C and KMT2D deficiencies result in aberrant activation of various signaling pathways, including the WNT pathway [98], as well as chromosomal instability [99,100].

Non-coding RNAs (ncRNAs), which represent over 90% of the total genome can be subdivided into long (>200 nucleotides) and small/short (<200 nucleotides) ncRNAs [90]. Long ncRNA can function as tumor suppressors or promotors in CRC, primarily by generating microRNAs (miRNAs) [101], a subtype of small ncRNAs containing 18–25 nucleotides [90]. miRNAs and other regulatory small ncRNAs play an integral role in gene expression [102], and consequently cancer cell proliferation, apoptosis, metastasis, and therapeutic resistance [102,103,104,105,106,107,108,109,110]. Aberrant miRNA expression and its role in CRC pathogenesis has been documented by other groups [103,111,112,113,114]. Recent studies have identified miRNA dysregulation in small bowel neuroendocrine tumors [115,116], however studies in SBA are lacking. Ongoing efforts focus on use of miRNA as a potential diagnostic and prognostic biomarker [102,105,106,107,108,109,110].

## 4. Conclusions and Future Directions

Genomic profiling has allowed for comprehensive characterization of the molecular landscape of SBA. Comparative analysis efforts have shown that SBA represents a unique molecular entity, with distinct gene alteration differences amongst small bowel subsites and neighboring gastrointestinal tumors. SBA remains a rare cancer without FDA approved therapies and treatment guidelines extrapolated largely from CRC. Recent understanding of the molecular drivers of SBA has identified potentially targetable genomic alterations in a large subset of patients. These targetable alterations should prompt further investigation to develop new therapeutic options that shape clinical management and establish new standards for a disease with limited therapeutic options and overall poor prognosis.
